# Case Report: Ureteral obstruction reveals rare underlying fibro-inflammatory, infiltrative, and proliferative disorders: report of four cases

**DOI:** 10.3389/fimmu.2026.1825897

**Published:** 2026-08-05

**Authors:** Luca Malatesta, Carlotta Rella, Francesco Pegoraro, Marco Allinovi, Maria Canfora, Edoardo Biancalana, Paola Romagnani, Ilaria C. Galli, Anna M. Buccoliero, Augusto Vaglio

**Affiliations:** 1 Department of Biomedical Experimental and Clinical Sciences “Mario Serio”, University of Florence, Florence, Italy; 2 Haematology and Oncology Department, Meyer Children’s Hospital Instituto di Ricovero e Cura a Carattere Scientifico (IRCCS), Florence, Italy; 3 Nephrology, Dialysis and Transplantation Unit, Careggi University Hospital, Florence, Italy; 4 Interdisciplinary Internal Medicine, Careggi University Hospital, Florence, Italy; 5 Nephrology and Dialysis Unit, Meyer Children’s Hospital Instituto di Ricovero e Cura a Carattere Scientifico (IRCCS), Florence, Italy; 6 Histopathology and Molecular Diagnostics, Careggi University Hospital, Florence, Italy; 7 Pathology Unit, Meyer Children’s Hospital Instituto di Ricovero e Cura a Carattere Scientifico (IRCCS), Florence, Italy

**Keywords:** amyloidosis AL, case report, Erdheim-Chester disease, Gardner syndrome, retroperitoneal fibrosis

## Abstract

**Introduction:**

Ureteral wall thickening or a periureteral “sleeve” on imaging often raise concern for malignancy, but rare non-carcinomatous disorder can mimic this presentation. We report radiology and pathology findings of four prototypical cases of ureteral obstruction due to rare diseases.

**Case presentation:**

We describe four cases presenting with nonspecific urological symptoms such as flank pain, hydronephrosis, and variable degrees of renal dysfunction, in whom computed tomography revealed segmental or circumferential periureteral thickening suggestive of an infiltrative process. Given the absence of specific clinical or laboratory findings, a biopsy was required to confirm the diagnosis in all cases. Four different and rare diseases were diagnosed, namely light-chain amyloidosis, idiopathic retroperitoneal fibrosis, Erdheim-Chester disease, and Gardner’s syndrome. Key imaging and pathology features are presented.

**Discussion and conclusions:**

Obstructive nephropathy may reveal rare fibro-inflammatory, infiltrative and proliferative diseases that require a thorough diagnostic work-up. Raising awareness of these rare conditions is crucial to guarantee timely diagnosis and effective treatment.

## Introduction

Ureteral and periureteral thickening or the presence of abnormal retroperitoneal tissue that infiltrates the ureters on imaging studies (*e.g.*, computed tomography [CT]), commonly raises suspicion for urothelial carcinoma or lymphoma. These imaging findings may appear in the context of diverse clinical presentations, ranging from flank pain to hematuria, hydronephrosis, or impaired kidney function. Rare, fibro-inflammatory or proliferative disorders can present with similar imaging features, making the diagnostic process considerably more complex (1). A limited number of reports in the last years have specifically addressed ureteral manifestations in these diseases, while even fewer have focused on their impact on renal function. As a result, these conditions may be misinterpreted as carcinomatous or lymphomatous processes on imaging, leading to diagnostic delays or unnecessary interventions. We here present four cases that highlight how a careful integration of clinical findings, imaging, and pathology is essential to accurately identify the underlying cause of obstructive nephropathy (2, 3). These case reports have been described following the CARE guidelines for case reports (CARE checklist available as Supplementary Material).

## Case presentation

In this report, we describe four patients who presented with nonspecific renal and urinary symptoms, including flank pain, unilateral or bilateral hydronephrosis, and variable degrees of renal dysfunction. In all cases, CT demonstrated abnormal periureteral tissue or ureteral wall thickening, segmental or circumferential. The lesions involved different ureteral segments, from lumbar to pelvic.

Routine laboratory tests, including tumour markers, were unremarkable, and the clinical presentations did not support a diagnosis of advanced urothelial carcinoma or lymphoma. This lack of diagnostic findings highlighted the importance of a comprehensive approach combining clinical evaluation, imaging, and, when necessary, tissue sampling. In our cases, histological confirmation was required to establish a definitive diagnosis.

Despite their similar radiologic appearance and clinical context, the histopathologic evaluation revealed four: localized AL (Amyloid Light-chain) amyloidosis, idiopathic retroperitoneal fibrosis (IRF), Erdheim-Chester disease (ECD), and desmoid disease related to Gardner’s syndrome. In all cases, the final diagnosis redirected management toward disease-specific therapy, thereby avoiding unnecessary oncologic treatments.

### Case 1

**Figure 1:**
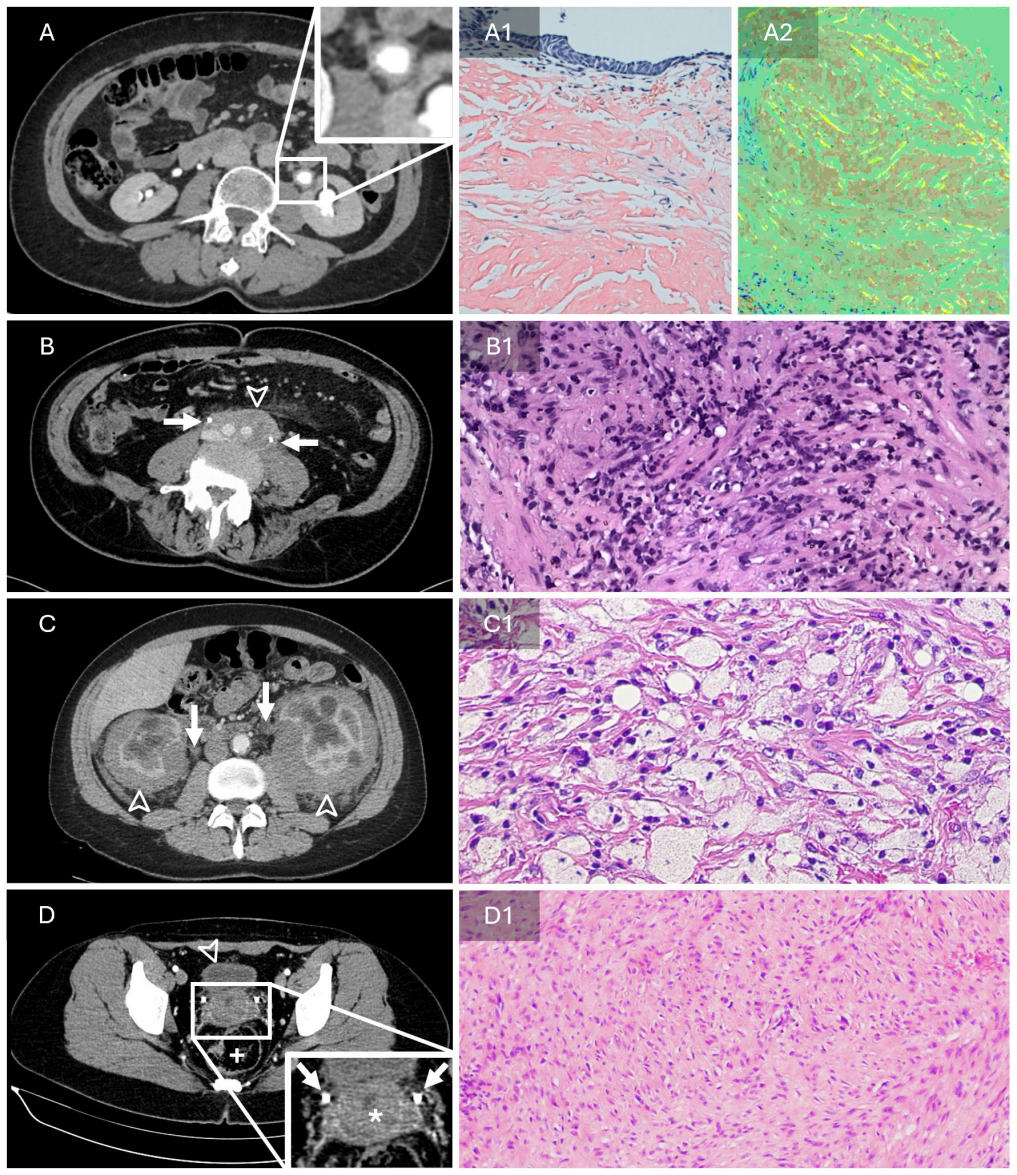
Radiologic and histopathologic features of rare disorders causing obstructive nephropathy. **(A, A1, A2)** Case 1 - Localized AL Amyloidosis. **(A)** Axial view of a contrast-enhanced abdominal CT showing a circumferential and homogeneously enhancing thickening of the left ureteral wall, with the boxed area enlarged in the inset. **(A1)** Excisional biopsy of the ureteral wall showing amorphous deposition which stains positive for Congo red (original magnification x20). **(A2)** Deposits exhibit apple-green birefringence under polarized light (original magnification x10). **(B, B1)** Case 2 - Idiopathic retroperitoneal fibrosis. **(B)** Axial view of a contrast-enhanced abdominal CT showing a solid retroperitoneal mass surrounding the common iliac arteries and encasing both ureters. White arrows indicate ureters. Arrowhead indicates retroperitoneal fibrosis. **(B1)** Hematoxylin-eosin (H&E) staining of the retroperitoneal biopsy showing dense fibrosis with interspersed lymphoplasmacytic infiltrates arranged in patchy aggregates, set within collagenous stroma (original magnification x20); immunohistochemistry revealed only fewer than 2 IgG4+plasma cells/High-Power Field with an IgG4/IgG ratio <5% (not shown). **(C, C1)** Case 3 - Erdheim-Chester disease. **(C)** Contrast-enhanced CT showing enlarged kidneys with “hairy kidney” appearance, typical of Erdheim-Chester disease. White arrows indicate the ureters, which are involved by the disease. Arrowheads indicate the peri-renal infiltration. **(C1)** H&E stain. The biopsy of the perirenal tissue shows a dense infiltrate of xanthomatous histiocytes with abundant vacuolated cytoplasms and small oval nuclei, set within fibrotic stroma and accompanied by chronic inflammatory infiltrates rich in lymphocytes (original magnification x20), immunohistochemistry showed positivity for CD68 and negativity for CD1a, S100 and langerin (not shown). **(D, D1)** Case 4 - Gardner’s Syndrome. **(D)** Contrasted enhanced CT showing enhancing plaque-like retrovesical tissue encasing both ureters; the boxed area is enlarged in the inset. White arrows indicate ureters. Asterisk (*) indicates the desmoid tumor. Arrowhead indicates the bladder. Plus sign (+) indicates the rectum. **(D1)** H&E stain. Long fascicles of bland fibroblasts and myofibroblasts with pale eosinophilic cytoplasm. Cells lack nuclear hyperchromasia, atypia, or mitotic figures (original magnification x4); immunohistochemistry demonstrated nuclear β-catenin positivity in lesional cells (not shown).

A 61-year-old woman with a family history (father) of transthyretin (TTR) cardiac amyloidosis was referred to our clinic for recurrent episodes of left-sided obstructive nephropathy and intercurrent acute kidney injury (AKI) with a serum creatinine of 1.2 mg/dL. Contrast-enhanced CT showed a circumferential and homogeneously enhancing thickening of the left ureteral wall, forming a solid periureteral cuff that extended over a long ureteral segment, resulting in hydronephrosis. This appearance raised a strong suspicion of an infiltrative ureteral malignancy, because of the concentric thickening and its enhancement (**Figure 1A**).

The patient underwent segmental ureterectomy. Histological examination of the surgical specimen revealed an inflammatory infiltrate with reactive changes of the urothelium, extensive red blood cell extravasation, and abundant and diffuse amorphous eosinophilic deposits within the *lamina propria*. This material was strongly Congo Red-positive and showed a characteristic apple-green birefringence under polarized light (**Figures 1A1, A2**), confirming amyloid deposition. Immunohistochemical analysis demonstrated reactivity for lambda light chains (with negative kappa light chains), also confirmed by immunogold electron microscopy (not shown). Additional testing, including immunostaining for tissue IgG4 and a complete systemic work-up, comprising serum and urine immunofixation, urinary Bence-Jones protein assessment, and free light chain measurement, were all negative or normal. On imaging and laboratory tests, including cardiac biomarkers (N-Terminal pro-B-type Natriuretic Peptide and high-sensitivity troponin), hepatic biomarkers (alkaline phosphatase and gamma-glutamyl transferase), and 24-hour urinary protein excretion for renal assessment, no other organs appeared to be involved by amyloidosis. There were no echographic features suggestive of cardiac amyloidosis. Genetic testing for TTR amyloidosis was negative. Based on the completely negative haematological screening and the absence of clinical, laboratory or imaging evidence of systemic amyloidosis, a bone marrow biopsy was not considered necessary. Abdominal fat pad biopsy was considered but not performed due to the lack of evidence of systemic amyloidosis and the expected low diagnostic yield in this setting. Therefore, we made a diagnosis of localized AL-type amyloidosis. The patient did not receive any treatment except for temporary ureteral stenting. After a follow-up of three years, her renal function steadily improved (serum creatinine 0.86 mg/dL, eGFR 77 ml/min/1.73 m²), and no evidence of systemic amyloidosis emerged.

### Case 2

A 72-year-old man with no relevant comorbidities was admitted to our department because of sudden-onset anuria. Laboratory tests showed severe AKI, with a serum creatinine of 15 mg/dL. Renal ultrasound revealed bilateral hydronephrosis. Contrast-enhanced abdominal CT identified a solid retroperitoneal tissue surrounding the abdominal aorta, the common iliac arteries and the inferior vena cava and encasing both ureters, leading to bilateral hydronephrosis (**Figure 1B**). Bilateral ureteral stents were inserted to relieve the obstruction. Positron emission tomography (PET)-CT scan showed intense fluorodeoxyglucose (FDG) uptake within the periaortoiliac soft tissue, while magnetic resonance imaging (MRI) demonstrated T2-weighted hyperintensity in the same region. Following stent placement, kidney function gradually improved, with serum creatinine decreasing to 1.7 mg/dL (eGFR 43 ml/min/1.73 m²).

A CT-guided core biopsy of the retroperitoneal tissue was then performed. Histology revealed a fibro-inflammatory tissue rich in mononuclear cells (**Figure 1B1**) but without significant amounts of CD138+ or IgG4+ plasma cells (immunohistochemistry showed fewer than 2 IgG4+plasma cells/High-Power Field with an IgG4/IgG ratio <5%; not shown). Serum IgG4 levels were within the normal range, and whole body-imaging (PET-CT and contrast-enhanced abdominal CT) together with physical examinations excluded the presence of other typical lesions belonging to the IgG4-Related Disease spectrum. Therefore, a diagnosis of IRF was made based on the typical radiological appearance, with CT scans showing involvement of the anterolateral aspect of the abdominal aorta. Secondary causes were systematically excluded through clinical history (no exposure to causative drugs, no previous radiotherapy, and no history of abdominal surgery), laboratory testing (negative Quantiferon-TB), and imaging findings. ECD, lymphoma, and other malignancies were considered unlikely based on the radiological presentation and were further excluded by histopathological examination of the biopsy specimen. Glucocorticoid therapy was started, later combined with mycophenolate mofetil as a steroid-sparing agent (4). Over the following months, the patient showed clear clinical and radiologic improvement, with a marked reduction in size of the periaortoliac and periureteral fibrotic mass. Serial imaging documented continuous regression of the fibro-inflammatory tissue and gradual resolution of hydronephrosis. One year after diagnosis, both ureteral stents were successfully removed without recurrence of obstruction. After a follow-up of three years, the patient remained clinically stable on low-dose prednisone and mycophenolate mofetil. Ultrasound and PET-CT confirmed sustained disease remission, with no evidence of metabolically active IRF, and kidney function remained stable (serum creatinine 1.5-1.6 mg/dL, eGFR 46–49 ml/min/1.73 m²).

### Case 3

A 50-year-old man with a history of hypertension and dyslipidemia came to our clinic for evaluation after bilateral hydronephrosis was incidentally detected on renal ultrasound, accompanied by a moderate kidney function impairment (serum creatinine 1.3 mg/dL, eGFR 67 ml/min/1.73 m²). On contrast-enhanced abdominal CT, both kidneys appeared enlarged and were surrounded by a thick hypodense tissue that enhanced with contrast (**Figure 1C**); this appearance was highly reminiscent of ECD, a systemic non-Langerhans cell histiocytosis. Bone scintigraphy was thus obtained and showed an increased symmetric uptake in the long bones, especially in the lower limbs, thus supporting the suspicion of ECD. Bilateral ureteral stents were placed to relieve obstruction. Routine blood tests and common neoplastic markers were within normal ranges. A percutaneous biopsy of the periureteral tissue was performed. Histological examination demonstrated a xanthomatous infiltrate composed of foamy histiocytes, consistent with ECD (**Figure 1C1**); immunohistochemistry showed positivity for CD68 and negativity for CD1a, S100 and langerin, supporting the diagnosis of ECD. The *BRAF^V600E^* mutation was not detected on the pathological tissue. A broader systemic evaluation, including whole-body CT, cardiac/Central Nervous System (CNS)/orbit MRI, and PET-CT, showed mild perirenal and periureteral disease but no other systemic involvement.

Based on this presentation and considering the therapeutic options available at the time of diagnosis (2014), treatment with everolimus was initiated based on the evidence emerging from a trial on mTOR inhibitors (5). The patient experienced a rapid improvement in pain, and his renal function stabilized, with serum creatinine around 1.7 mg/dL and eGFR of 48 ml/min/1.73 m². Follow-up FDG-PET-CT demonstrated a clear reduction in radiotracer uptake in the perirenal soft tissue, suggesting a favourable response to therapy. At the latest follow-up, 12 years after diagnosis, the patient continued to show stable disease, preserved renal function, and no evidence of additional organ involvement. Given the sustained response to mTOR inhibition and the absence of disease progression, additional molecular testing (e.g., for other somatic mutations in the MAPK pathway) was not performed on the diagnostic biopsy; and targeted therapies was not pursued.

### Case 4

A 31-year-old woman with a known diagnosis of familial adenomatous polyposis (FAP), genetically confirmed through detection of a pathogenic *APC* mutation, had undergone total colectomy at the age of 25 years. Her past medical history included Raynaud’s phenomenon, episodes of oral aphthosis, and active smoking. Her father also had FAP, had undergone colectomy, and later developed a desmoid tumor (the association between FAP and desmoid tumors is referred to as Gardner’s syndrome). Although desmoid tumors are benign, they may have a locally aggressive behaviour (6).

The patient was admitted for urinary sepsis and was found to have bilateral hydronephrosis. She also reported weight loss, persistent fever, gross hematuria, and flank pain. Kidney function at that time was normal (serum creatinine 0.8 mg/dL, eGFR 101 ml/min/1.73 m²). Bilateral ureteral stents were placed to relieve obstruction. PET-CT revealed two pseudo-focal hypermetabolic areas within the mesenteric fat in a pre-vertebral location. Abdominal CT demonstrated an enhancing plaque-like tissue formation surrounding both ureters; the lesion was more pronounced on the right side, where it extended for nearly 10 cm at the lumbosacral level (**Figure 1D**). A biopsy of the lesion was performed, revealing bland fibroblastic and myofibroblastic proliferation without atypia or mitotic activity (**Figure 1D1**). Immunohistochemical analysis demonstrated nuclear β-catenin positivity in lesional cells, supporting the diagnosis of desmoid-type fibromatosis. In the context of genetically confirmed FAP, the clinical, imaging, and histopathologic findings were consistent with an intra-abdominal desmoid tumor associated with Gardner syndrome, involving the mesentery and the retroperitoneum and encasing both ureters. The timing of presentation was also compatible with desmoid disease in the context of FAP.

At the time of diagnosis, tamoxifen (7) was a commonly used systemic treatment option for desmoid tumors, particularly in symptomatic patients with intra-abdominal disease. Given the presence of bilateral ureteral obstruction and the need to reduce tumor-related activity, tamoxifen 20 mg/day was initiated after discussion of available therapeutic options. According to current consensus, active surveillance represents the preferred initial approach for most patients with desmoid-type fibromatosis, with systemic therapy reserved for progressive, symptomatic, or function-threatening disease, particularly in intra-abdominal locations. Among systemic options, gamma secretase inhibitor (as Nirogacestat) (8) has demonstrated significant improvement in progression-free survival and objective response in the phase III DeFi trial and received FDA approval in 2023 (9) for adults with progressing desmoid tumors requiring systemic therapy. Tyrosine kinase inhibitors (10) represent additional therapeutic alternatives in selected cases. Five months later, both ureteral stents were removed. Follow-up ultrasound showed improvement of the previously observed hydronephrosis. During subsequent years, serial ultrasound examinations confirmed a stable reduction of the ureteral dilation, and PET-CT showed no residual hypermetabolic activity. Renal function remained stable, with serum creatinine unchanged from baseline. The treatment was well tolerated and continued for 7 years, with no significant adverse effects.

## Discussion and conclusions

Periureteral thickening or retroperitoneal lesions involving the ureters often suggest an underlying carcinomatous or lymphomatous disease. However, as shown in these cases, the radiologic and histopathologic findings may disclose rare conditions. Although imaging findings were highly suggestive, the overlapping radiological features of retroperitoneal diseases, urothelial carcinoma, and lymphoma required histological confirmation to establish a definitive diagnosis. The cases we present here are prototypical examples of four distinct and quite rare conditions that may cause ureteral obstruction, namely localized AL amyloidosis, IRF, ECD and desmoid tumor in the context of Gardner’s syndrome.

Localized AL amyloidosis of the urinary tract might present with focal or diffuse ureteral thickening that resembles urothelial carcinoma (11). Clinical manifestations, including hematuria and obstructive symptoms, are nonspecific, and imaging alone cannot exclude this condition. In most cases, the diagnosis relies on histological confirmation; serum monoclonal gammopathy is often absent (12, 13). Another entity that may cause ureteral obstruction and may mimic urothelial carcinoma or lymphoma is IRF, a rare fibro-inflammatory disease that often arises in the context of IgG4-Related Disease (14). Its clinical presentation overlaps with that of retroperitoneal malignancies, and it is therefore frequently misinterpreted as a malignant process on initial evaluation (15). Radiological findings such as a homogeneous soft-tissue density mass located around the antero-lateral sides of the infrarenal abdominal aorta and the iliac arteries, with medial ureteral deviation or obstruction and frequent inferior vena cava involvement represents a hallmark of IRF (16). ECD, a rare systemic histiocytosis, frequently involves the retroperitoneum and the periureteral space, usually the proximal ureters (as opposed to IRF, which instead entraps the distal ureters) (17). The xanthomatous and fibrotic infiltration typical of ECD can mimic an infiltrative tumor. Characteristic imaging features, such as the “hairy kidney” appearance, may not always be present (18). Finally, Gardner’s syndrome, an autosomal dominant condition caused by *APC* gene mutations, presents with intestinal polyposis and desmoid tumors (19). Although benign, desmoid tumors are locally infiltrative. When they arise close to the ureters, they may encase or compress them, reproducing the radiological appearance of an infiltrative carcinoma or lymphoma (20). All of these conditions, although of different origin, share clinico-pathological features such as a slow clinical course, an intense fibrous or fibro-inflammatory reaction, and, in some cases, association with systemic disease. In practice, periureteral or retroperitoneal lesions that show a slow progression, unusual imaging features, or systemic involvement should prompt consideration of diagnoses other than carcinoma or lymphoma. Systemic features are particularly important in this respect: for example, findings such as infiltration of different sites such as the soft tissues of the eye, the salivary or lacrimal glands, mediastinum, periaortic space, and pancreato-biliary system should heighten suspicion of an IgG4-Related Disease (21). On the other hand, diffuse bone involvement, simultaneous periaortic and perirenal disease, as well as other features such as xanthelasma or cardiac involvement are evocative of ECD (22).

In conclusion, the cases presented here highlight that several different rare lesions that cause ureteral thickening or retroperitoneal lesions with ureteral obstruction may mimic urothelial carcinoma or lymphoma. Accurate differentiation is essential as management strategies differ substantially. A thorough clinico-radiologic, histopathologic and sometimes genetic work-up is necessary to reach the diagnosis (as resumed in **Table 1**). The presence of systemic organ involvement must be accurately investigated. The main limitations of this study include the small number of cases and the descriptive, retrospective nature of this case series.

**Table 1:** Structured summary of the four cases included in this case report. For each patient, demographic characteristics, presenting symptoms, key laboratory findings, CT features, histopathological findings, final diagnosis, treatment, and clinical outcome with follow-up duration are reported in parallel columns to allow direct comparison.

Case	Demographics	Clinical presentation	Key laboratory findings	CT features	Biopsy	Diagnosis	Treatment	Outcome/follow-up duration
1	Woman, 61y.o.	Flank pain	Creatinine 1.2 mg/dL; IgG4, serum-urine immunofixation, urinary Bence-Jones protein, free light chain → normal	Circumferential enhancing and thickening of the left ureteral wall causing hydronephrosis	Diffuse amorphous eosinophilic deposits within the lamina propria; Congo Red–positive amyloid deposits with apple-green birefringence; lambda light-chain restriction	Localized AL-type amyloidosis	Segmental ureterectomy + temporary stent	Renal function improved (Creatinine 0.86 mg/dL); 3-year follow-up
2	Man, 72 y.o.	Anuria	Creatinine 15 mg/dL; IgG4 → normal	Retroperitoneal tissue encasing aorta and both ureters with bilateral hydronephrosis	Fibro- inflammatory tissue without significant CD138+ or IgG4+ plasma cells	Idiopathic retroperitoneal fibrosis	Temporary bilateral stents + glucocorticoids + mycophenolate mofetil	Clinical/radiologic improvement; Creatinine 1.5-1.6 mg/dL; 3-year follow-up
3	Man, 50 y.o.	Asymptomatic	Creatinine 1.3 mg/dL	Bilateral enlarged kidneys with perirenal enhancing hypodense tissue (“hairy kidney”)	Xanthomatous histiocytic infiltrate consistent with ECD; CD68+, CD1a-, S100-, langerin-; BRAF^V600E^ negative	Erdheim-Chester disease	Temporary bilateral stents + Everolimus	Creatinine 1.7 mg/dL; 12-year follow-up
4	Woman, 31 y.o.	Weight loss, fever, gross hematuria, flank pain	Creatinine 0.8 mg/dL	Plaque-like enhancing tissue surrounding both ureters	Bland fibroblastic/myofibroblastic proliferation without atypia; nuclear β-catenin positivity.	Gardner’s syndrome	Temporary bilateral stents + tamoxifene	Clinical/radiologic improvement; creatinine 0.8 mg/dL; 7-year follow-up

## Patients’ perspectives

All patients described the diagnostic phase as emotionally challenging, mainly because imaging findings strongly suggested a possible urothelial carcinoma or lymphoma, generating significant fear of certain types of cancer. The period of uncertainty before histological confirmation was perceived as highly distressing. Once a definitive diagnosis of a fibro-inflammatory, infiltrative or proliferative condition was established, patients reported substantial relief. Nevertheless, they also expressed anxiety related to receiving diagnoses of rare and largely unfamiliar diseases, as well as concerns about undergoing treatments that are not widely known or understood outside the medical community. After treatment initiation, however, the absence of significant short- or long-term adverse effects, together with the observed clinical and radiologic benefits, contributed to increased confidence in the therapeutic strategy, improved adherence, and a renewed sense of trust and reassurance regarding their prognosis.

## Data Availability

The raw data supporting the conclusions of this article will be made available by the authors, without undue reservation.
